# Analysis of Pregnant Women Recovered From Antenatal SARS-CoV-2 Infection: An Observational Study

**DOI:** 10.7759/cureus.22094

**Published:** 2022-02-10

**Authors:** Mousumi D Ghosh, Mamta R Datta, Vinita Singh, Anisha Choudhary

**Affiliations:** 1 Obstetrics and Gynaecology, Tata Main Hospital, Jamshedpur, IND

**Keywords:** covid-19, antenatal, outcome, recovery, pregnancy, sars-cov-2

## Abstract

Background and objective

The global health care system is facing the challenge of diagnosing and treating the ongoing coronavirus disease 2019 (COVID-19) pandemic. Pregnant women belong to a vulnerable group, and the effect of the virus on the mother and fetus is not well established. The aim of the study was to understand the maternal and fetal outcomes after recovery from antenatal severe acute respiratory syndrome coronavirus 2 (SARS-CoV-2).

Methods

This was a retrospective observational study conducted at Tata Main Hospital, Jamshedpur, India. It included all COVID-19-negative pregnant women who had delivered between 1^st^ January 2021 and 31^st ^August 2021 and had tested positive in the antenatal period (by reverse transcription-polymerase chain reaction (RT-PCR)), the details of which are available in the hospital database.

Results

A total of 53 women were included in our study who had tested positive in the antenatal period and had turned negative during delivery. Out of the 53 women, 5.7% were infected in the first trimester, 34% in the second trimester, and 60.3% were positive in the third trimester. We found an asymptomatic subgroup in 52.8% of women and mild symptoms in 41.5% of women. Two women were admitted in their antenatal period with moderate COVID-19 disease and one with severe. Preterm births between 34 weeks and 37 weeks were seen in 26.4% of women. Vaginal delivery accounted for 30.2% of cases. The most common indications for cesarean section were fetal distress (17%), previous cesarean section (17%), and unwillingness for vaginal delivery. Out of the 53 pregnant women included in the study, acute respiratory distress syndrome (ARDS) was seen in two women- one diagnosed intraoperatively during cesarean section and the other was diagnosed on the first postoperative day.

Conclusion

The study showed that pregnant women infected with SARS-CoV-2 usually have no/mild symptoms, and they recover well and have favorable maternal and neonatal outcomes. However, perinatal vigilance is advisable in these cases, as there is a risk of developing respiratory morbidity.

## Introduction

The coronavirus disease 2019 (COVID-19) pandemic is one of the most devastating crises of our times. It is caused by severe acute respiratory syndrome coronavirus 2 (SARS-CoV-2). Globally, the health care system is facing the challenge of diagnosing and treating this novel viral disease. Knowledge about the effect of COVID-19 disease on pregnancy is limited. Few studies show increased risks of adverse outcomes due to pregnancy-induced immune suppression while others suggest protective phenomena due to physiologic responses [1].

There are very few studies on pregnancy outcomes in women with a history of COVID-19 infection during their antenatal period with recovery before delivery. In the present study, we have studied the maternal and neonatal outcomes of pregnant women who were infected with severe acute respiratory syndrome coronavirus 2 (SARS-CoV-2) antenatally.

## Materials and methods

This was a retrospective observational study of pregnant women admitted to the department of obstetrics and gynecology, at Tata Main Hospital, a tertiary care hospital in eastern India from 1st January to 31st August 2021. It is a designated COVID-19 care hospital with the largest COVID-19 labor room and OPD services (physical and telephonic) in the city.

All COVID-19-negative (by reverse transcription-polymerase chain reaction (RT-PCR)) pregnant women admitted to hospital between 1st January 2021 and 31st August 2021 who had a prior confirmed COVID-19 infection during the present pregnancy were included in our study. The previous positive reports and details of treatment (outdoor consultation/ indoor admission) were confirmed from the Hospital Management System (hospital database of patients). 

Data were entered on Microsoft Excel sheets (Microsoft Corporation, Redmond, WA). Details about their age, parity, period of gestation when infected, symptoms, associated comorbidities, and treatment received were collected. The delivery details, along with maternal and fetal outcomes, were tabulated. All categorical variables are expressed as frequency and percentages.

## Results

A total of 1844 pregnant women were admitted for confinement in the department of obstetrics and gynecology from 1st January 2021 to 31st August 2021. Among the COVID-19-negative women, 53 (2.8%) tested positive in their antenatal period, and their details were available in the hospital database.

The demographic profile of the patients is depicted in Table 1. The age of the patients ranged from 18 years to 48 years and the maximum number of patients were between 21 to 30 years ago. Primigravida constituted 47.2% and the rest were multigravida. Only 5.7% of patients were infected in the first trimester. Thirty-four percent (34%) of patients were positive in the second trimester and 60.3% were positive in the third trimester.

**Table 1:** Demographic profile of the patients

Parameters	Number of patients(n)	Percentage(%)
Age group (years)		
<20	3	5.7
21-30	30	56.6
31-40	19	35.9
>40	1	1.8
Total	53	100
Parity		
Primigravida	25	47.2
Multigravida	28	52.8
Total	53	100
Gestational age when infected (weeks)		
<12	3	05.7
12-28	18	34.0
>28	32	60.3
Total	53	100

None of these patients received COVID-19 vaccination. The Ministry of Health and Family Welfare, Government of India, approved vaccination of pregnant women against COVID-19 on 2nd July 2021.

The symptoms and maternal co-morbidities are listed in Table 2. We have classified the symptoms according to clinical findings [2]. Asymptomatic patients are those with positive SARS-CoV-2 testing but no symptoms. Mild symptoms are those with fever, cough, headache but no dyspnea or abnormal imaging. Moderate symptoms are those with clinical or radiographic evidence of mild pneumonia/ oxygen saturation >93% on room air at sea level. Severe disease includes those with dyspnea (respiratory rate >30/minute), oxygen saturation less than 93%, or imaging showing more than 50% lung infiltrates.

**Table 2:** Symptoms and maternal comorbidities

Parameters	Number of patients (n)	Percentage(%)
Symptoms of COVID-19 infection during antenatal infection		
Asymptomatic	28	52.8
Mild	22	41.5
Moderate	2	03.8
Severe	1	01.9
Total	53	100
Hospitalization during antenatal COVID-19 infection		
COVID-related cause	3	05.7
Obstetric cause	6	11.3
Not admitted	44	83
Total	53	100
Maternal comorbidities		
Anemia	4	07.5
Diabetes mellitus/gestational diabetes mellitus	15	28.3
Chronic hypertension/gestational hypertension	9	17.0
Deranged liver enzymes	12	22.6
Thrombocytopenia	2	03.8
Hypothyroidism	4	07.5
Body mass index >30	4	07.5
Multifetal pregnancy	3	05.7
Total	53	100

In our study, we found an asymptomatic subgroup in 52.8% of patients and mild symptoms in 41.5% of patients. There were two patients with moderate and one with severe symptoms, respectively, who needed admission for COVID-19-related causes. Six patients needed admission for obstetric causes, but they were asymptomatic for COVID-19. Testing was done in these asymptomatic groups as part of universal testing for all admissions. These two groups of patients (asymptomatic and mild category) had access to a telephonic consultation with a physician and obstetrician as and when needed [3]. Those with mild infection were advised home isolation and to take paracetamol tablets round the clock for fever along with multivitamins. They were suggested to maintain adequate hydration. The indications for emergency consultation were explained to them. Further follow-up and monitoring of maternal and fetal well-being were done as per the Indian Council of Medical Research and the National Institute for Research in Reproductive Health guidelines [3-4].

Among the maternal co-morbidities, 7.5% were anemic and half of these patients needed a blood transfusion. Diabetes mellitus, hypertensive disorders, and deranged liver enzymes were found in 28.3%, 17%, and 22.6% of patients, respectively.

The mothers were subsequently followed up in antenatal clinics and a fetal growth scan was done in all cases. The delivery details are elaborated in Table 3. Preterm births were found in 26.4% of patients; these were between 34 and 37 weeks of pregnancy. Maximum patients (37.8%) delivered between 37 and 38 weeks, and there was no childbirth beyond 40 weeks of pregnancy. Vaginal delivery accounted for 30.2% of cases. The most common indications for cesarean section were fetal distress (17%) and previous cesarean section (17%), not willing for vaginal delivery.

**Table 3:** Delivery details and maternal outcome FGR: Fetal Growth Retardation; ARDS: Acute Respiratory Distress Syndrome

Parameters	Number of patients(n)	Percentage(%)
Time of delivery (weeks)		
Preterm birth (<34)	0	0
Preterm birth (34-37)	14	26.4
37-38	20	37.8
38-39	8	15.1
39-40	11	20.7
>40	0	0
Total	53	100
Mode of delivery		
Vaginal delivery	14	26.4
Instrumental vaginal delivery	2	03.8
Lower segment cesarean section	37	69.8
Total	53	100
Indication of lower segment cesarean section		
Fetal distress	9	17.0
Previous cesarean section	9	17.0
Failed induction of labor	6	11.3
Not willing for trial of labor	5	9.4
FGR with severe oligohydramnios	4	07.5
Non-progress of labor	2	03.8
Abnormal presentation/placentation	2	03.8
Total	37	100
Perinatal maternal morbidity		
ARDS	2	3.8
Anemia needing a blood transfusion	2	3.8
Total	53	100

Induction of labor was required in 14 (26.4%) women, mainly for gestational hypertension, less liquor, and obstetric cholestasis. Out of these, eight women had a vaginal delivery and six women needed cesarean section. The indications for cesarean section after induction of labor were fetal distress, non-progress of labor, and failed induction.

Acute respiratory distress syndrome (ARDS) was seen in two cases during childbirth - one diagnosed intraoperatively during cesarean section and the other was diagnosed on the first postoperative day. The first patient was a case of twin pregnancy, a previous cesarean section with gestational hypertension who tested positive at 28 weeks of pregnancy with mild symptoms. She recovered with oral antibiotics and multivitamins. She had preterm premature rupture of membranes (PPROM) and cesarean delivery at 35 weeks two days under spinal anesthesia. Intraoperatively, she developed breathlessness and pulmonary edema, treated with diuretics. She recovered after non-invasive ventilatory support of two days and was discharged on postoperative day four. The other patient had tested COVID-19 positive at 29 weeks of pregnancy, had moderate symptoms, and needed admission. Induction of labor was done at 37 weeks two days for fetal growth retardation and less liquor. She had an emergency cesarean section for fetal distress. On the first postoperative day, she developed basal lung crepitation and a chest X-ray showed increased broncho-vascular markings. The cardiac evaluation was found normal. She was discharged on day five after recovery and the baby's discharge from the nursery. Both these patients were managed with a multidisciplinary team of physicians, intensivists, and anesthetists along with obstetricians [5]. Two patients with iron deficiency anemia needed a blood transfusion in the postoperative period. There was no maternal death.

The details of neonatal outcomes are in Table 4. The weight of babies ranged from 1.5 kg to 3.7 kg and 47.1% of them weighed between 2.5 to 2.9 kg. Thirty-four percent (34%) of babies needed nursery admission, reasons being low birth weight, blood sugar monitoring in diabetic mothers, and neonatal jaundice. All these babies were discharged in good health. No babies had pneumonia, and there were no neonatal deaths [6].

**Table 4:** Neonatal outcomes NICU: Neonatal Intensive Care Unit

Parameters	Number of neonates(n)	Percentage (%)
Birth weight of new-born (kilogram)		
< 1	0	0
1.0-1.4	0	0
1.5- 1.9	4	07.5
2.0-2.4	9	17.0
2.5-2.9	25	47.1
3.0-3.4	12	22.6
3.5-3.9	3	05.7
>4	0	0
Total	53	100
Neonatal details		
Nursery admission (total)	18	34.0
NICU admission for low birth weight	5	27.77
NICU admission for neonatal blood sugar monitoring	8	44.44
NICU admission for neonatal jaundice	5	27.77
Neonatal death	0	0

## Discussion

Severe acute respiratory syndrome coronavirus 2 (SARS CoV-2) is a new challenging disease. Pregnancy is a vulnerable group and a matter of great concern, as its effect on the mother and fetus is not known. Women experience immunologic, hormonal, and metabolic changes during pregnancy. In this study, we included pregnant women who were infected with SARS-CoV-2 during their antenatal period but recovered before delivery. The purpose of this study was to understand the clinical manifestations of COVID-19 in pregnancy, the effects of pregnancy on the course of COVID-19 disease, and the pregnancy outcome after recovery.

In our study, 52.8% of patients were asymptomatic. They were tested on admission due to obstetric causes, travel history as per state guidelines, and high-risk contact of positive patients. Forty-one point five percent (41.5%) of patients had mild symptoms and recovered with advice on an outpatient basis. Two patients with moderate disease and one with severe disease needed admission for COVID-19 illness, accounting for 5.7%. In a study by Adhikari et al [7], 6% of patients needed admission for COVID illness. Our patient with moderate disease tested positive at 33 weeks three days and needed admission. She had induction of labor at 37 weeks two days gestation for cholestasis of pregnancy and had a vaginal delivery with no maternal and neonatal morbidity. Another patient with moderate disease tested positive at 28 weeks. She had induction of labor at 37 weeks one day for less liquor. She had an emergency cesarean section for fetal distress and developed ARDS postoperatively. The patient with severe disease was a 33-year-old case of in-vitro-fertilization (IVF) with gestational diabetes mellitus (GDM) who received injection remdesivir [8] after informed consent. She needed oxygen support, steroids, anticoagulants, and antibiotics. She had a cesarean section at 37 weeks for in-vitro-fertilization (IVF) pregnancy, not being willing for the trial of labor. The mother had an uneventful peripartum period, however, the baby needed nursery admission for blood sugar monitoring, as she was a case of gestational diabetes mellitus. No patients needed admission to the intensive care unit (ICU).

Hospitalization during an antenatal COVID-19 infection was 17%, which included COVID-related as well as obstetric causes. Studies suggest that SARS-CoV-2 can cause miscarriages [9], stillbirth, and preterm delivery [10-11]. Miscarriages could not be studied here, as we included mothers who came for delivery in the third trimester. In our study, only 5.7% of patients were infected in the first trimester, however, they did not have any maternal or neonatal morbidity. There was no stillbirth in our study. There was no delivery before 34 weeks of pregnancy and 26.4% of patients delivered between 34 and 37 weeks. A systematic review of 33 studies showed a preterm birth rate of 15.2% [11].

Sixty point three percent (60.3%) of women tested positive in the third trimester of pregnancy in our study. In a study by Siddiqui et al, 74% were infected in the third trimester and the most common age group was between 21 and 30 years [12]. A study by Qeadan et al. pointed out that COVID-19 positive mothers are not at increased risk for invasive ventilation or death [13].

Gestational diabetes, hypertension, and deranged liver enzymes were the most common morbidities in our study. The cesarean section rate was 74% according to Anuk et al. [14], whereas it was 69.8 % in our study [13]. The mothers had a lower incidence of spontaneous onset of labor [15].

The strength of our study is the accuracy of information as the data collected during delivery is verified with information on hospital database management against the specific registration number of the patient. Also, none of the patients received COVID-19 vaccination, which would affect the course of the disease.

## Conclusions

The study showed that pregnant women infected with SARS-CoV-2 recover well and have favorable outcomes. Pregnant women are not at risk of severe disease. The pregnancy continues to term with good maternal and fetal outcomes. However, perinatal vigilance is advisable in these cases, as there is a risk of developing respiratory morbidity. However, studies on follow-up of these mothers and neonates are required to assess any long-term effects or complications.
